# Jet-associated resonance spectroscopy

**DOI:** 10.1140/epjc/s10052-017-5416-2

**Published:** 2017-12-07

**Authors:** Christoph Englert, Gabriele Ferretti, Michael Spannowsky

**Affiliations:** 1 SUPA, School of Physics and Astronomy, University of Glasgow, Glasgow, G12 8QQ UK; 2 Department of Physics, Chalmers University of Technology, Fysikgården, 41296 Göteborg, Sweden; 3 Department of Physics, Institute for Particle Physics Phenomenology, Durham University, Durham, DH1 3LE UK

## Abstract

We present a model-independent study aimed at characterising the nature of possible resonances in the jet-photon or jet-*Z* final state at hadron colliders. Such resonances are expected in many models of compositeness and would be a clear indication of new physics. At leading order, in the narrow width approximation, the matrix elements are parameterised by just a few constants describing the coupling of the various helicities to the resonance. We present the full structure of such amplitudes up to spin 2 and use them to simulate relevant kinematic distributions that could serve to constrain the coupling structure. This also generalises the signal generation strategy that is currently pursued by ATLAS and CMS to the most general case in the considered channels. While the determination of the P/CP properties of the interaction seems to be out of reach within this framework, there is a wealth of information to be gained about the spin of the resonance and the relative couplings of the helicities.

## Introduction

Many scenarios of dynamics Beyond the Standard Model (BSM), built with the aim to ameliorate the hierarchy problem, predict the existence of new resonances at the TeV scale. Examples of such states abound in many different contexts such as vector-like confinement [1, 2], compositeness [3–10], partial compositeness [11–16] and excited quarks [17–22].

If such states are indeed observed in the future at the LHC (or at a future hadron collider), the first step in obtaining a detailed picture of the underlying theory is a dedicated spectroscopy program targeting the nature of their couplings, spin and CP properties.

These new resonances do not necessarily have to be QCD singlets and, thus, the general spectroscopy program that has been pursued in conjunction to the Higgs discovery [23–30], using the pioneering techniques of [31] needs to be augmented by considering jet-inclusive final states.

While di-jet analyses exist and are already used to constrain the presence of BSM physics, analyses of electro-weak bosons in association with jets have received less attention (but do exist as well; see e.g. [32–35]). This is predominantly due to the fact that these channels are less common in established BSM scenarios and limits are typically dominated by cleaner *ZZ* or $\gamma \gamma$ channels. However, particularly in the aforementioned scenarios of (partial) compositeness, these channels do provide important information as regards the couplings of a possible discovery. This motivates searches and a characterisation program of jet-$\gamma$ and jet-*Z* resonances (related by gauge invariance) as an important probe of BSM physics at the LHC. Conversely, the lack of an observation in these channels would help to restrict the parameter space for such models. In either case, a detailed understanding of the possible dynamical scenarios is needed to perform an efficient analysis.

From a phenomenological perspective, jet-associated resonances are appealing as they combine potentially large signals, due to the presence of coloured particles, with the precision of having a highly energetic photon or final state leptons, thereby being well covered by existing trigger requirements. Searches so far have not given any hint for such resonances. However, much more data is being collected and hopefully will soon be analyzed. We expect that studies of jet-$\gamma$ resonances will feature among the many channels in which searches for BSM physics are being carried out.

The purpose of this note is to present a model-independent leading-order analysis of the various possibilities of jet-$\gamma /Z$ interactions, aimed at extracting information as regards the spin of such resonances from the kinematic distribution of the outgoing photon or reconstructed *Z* boson. (It is important to stress that EFT-based power-counting arguments might not be valid in case of a strongly interacting nature of such a resonance.)

In Sect. 2, we analyze the cases of spin $j=0, \frac{1}{2}, 1, \frac{3}{2}, 2$, and construct the general form for the model-independent amplitude at parton level. In Sect. 3, we bridge these amplitudes to the hadron level for chosen benchmark scenarios and point out the sensitivity that can be expected from a dedicated spectroscopy program in these channels.

## Model-independent amplitudes

In this section we present the amplitudes relevant for the study of jet-$\gamma$ and jet-*Z* resonances. In the narrow width approximation, at leading order, we can decompose the amplitude into $2\rightarrow 1$ (production) and $1\rightarrow 2$ (decay) on-shell processes, each characterised by a handful of coefficients coupling the different helicities.

For the jet-$\gamma$ case, these coefficients are denoted by $a^{\mathcal {P}\mathcal {P}'}$ and $b^{\mathcal {P}\mathcal {P}'}$, where $\mathcal {P}$ and $\mathcal {P}'$ are the relevant “partons” and *b* refers to the larger helicity component (below we will also label these coefficients with a subscript indicating the spin of the resonance). The P/CP properties are indicated by putting a tilde on those coefficients related to amplitudes containing a $\gamma ^5$ or a $\epsilon$ tensor.

We retain the same notation for the jet-*Z* case, so that amplitudes with the same *a* or *b* coefficients reduce to the previous ones in the $m_Z\rightarrow 0$ limit. The additional coefficients arising from the longitudinal modes of the *Z* are denoted by $c^{\mathcal {P}\mathcal {P}'}$ and the corresponding amplitudes vanish in the $m_Z\rightarrow 0$ limit.

Since all $2\rightarrow 1$ and $1\rightarrow 2$ amplitudes have the dimension of a mass, we divide by the appropriate power of the resonance mass *M* to get the right overall dimension so that all coefficients *a*, *b*, *c* are dimensionless. In the case where these coefficients arise from an effective field theory at the scale $\Lambda \gg M$, they will then scale by the appropriate powers of $M/\Lambda$ but we find it unnecessary to introduce an additional scale in the kinematics at this stage.

### The jet$+\gamma$ case

We begin by looking at $2\rightarrow 1$ processes involving 2 incoming massless particles of spin $1 \text { or }\frac{1}{2}$ creating a resonance of spin $0, \frac{1}{2}, 1, \frac{3}{2}$ or 2. These amplitudes are those of relevance to the production of the resonance but also, by using CPT, to the decay into a parton and a photon. In the next subsection we will consider the inclusion of the *Z* boson.

For the process $\mathcal {P}_1 \mathcal {P}_2 \rightarrow X$ we introduce the partons’ on-shell four-momenta, generically denoted by $p_1$ and $p_2$, ($p_1^2 = p_2^2 = 0$) as well as $p=p_1 + p_2$ and $q=p_1 - p_2$, obeying $p^2 = - q^2 = 2 p_1\cdot p_2= M^2$. The gluon and photon four-dimensional polarisation vectors are transverse and the quark spinors obey the massless Dirac equation with the associated momenta.

As far as the polarisations of the resonance *X* are concerned, we have  and  for spin $\frac{1}{2}$; $p^\mu S_\mu = 0$ for spin 1;  and  for spin $\frac{3}{2}$; $S_{\mu \nu } = S_{\nu \mu }, S_\mu ^\mu = 0$ and $p^\mu S_{\mu \nu }=0$ for spin 2.

It is then straightforward to construct all combinations that are Lorentz invariant and obey the Ward identities. What is a bit more tedious is to eliminate all the linearly dependent combinations, particularly for the case containing the $\epsilon$-tensor where one needs to use Schouten’s identity. This computation is greatly simplified by going to the centre-of-mass frame (cms) of the resonance.

We always assume flavour conservation, so the quark polarisations *u*, *v* refer to the same quark flavours. The colour indices of the resonance are denoted by *A* (octet) or *a* (triplet/anti-triplet), those of the partons by $B, B', b$ or $b'$. In the case of two gluons we denote by $\varepsilon$ and $\varepsilon '$ their polarisations and we can use either the totally anti-symmetric $f^{ABB'}$ or the totally symmetric $d^{ABB'}$ to respect the overall Bose symmetry, rendering selections rules a-la’ Landau–Yang irrelevant in this case (a fact also mentioned in [36]). The amplitudes read as follows.

Spin 0:

$$\begin{aligned}&\gamma ~g\rightarrow X:\ \delta ^{AB}\left( a^{\gamma g}_0 \left( M \varepsilon ^{(\gamma )}_\mu {\varepsilon ^{(g)}}^\mu + 2 \varepsilon ^{(\gamma )}_\mu q^\mu \varepsilon ^{(g)}_\nu q^\nu /M\right) \right. \nonumber \\&\quad \left. +\,\tilde{a}^{\gamma g}_0 \varepsilon ^{(\gamma )}_\mu \varepsilon ^{(g)}_\nu q_\lambda p_\rho \epsilon ^{\mu \nu \lambda \rho }/M\right) , \nonumber \\&g~g\rightarrow X:\ d^{ABB'} \left( a^{g g}_0 \left( M \varepsilon _\mu {\varepsilon '}^\mu + 2 \varepsilon _\mu q^\mu \varepsilon '_\nu q^\nu /M\right) \right. \nonumber \\&\quad \left. +\,\tilde{a}^{g g}_0 \varepsilon _\mu \varepsilon '_\nu q_\lambda p_\rho \epsilon ^{\mu \nu \lambda \rho }/M\right) , \nonumber \\&q~\bar{q}\rightarrow X:\ T^{Ab}_{b'} \left( a^{q\bar{q}}_0 \bar{v} u + i \tilde{a}^{q\bar{q}}_0 \bar{v} \gamma ^5u\right) . \end{aligned}$$

Spin $$\frac{1}{2}$$:

$$\begin{aligned}&\gamma ~q\rightarrow X:\ \delta ^a_b \left( \frac{1}{\sqrt{2}} a^{\gamma q}_{1/2}( \varepsilon _\mu \bar{U} \gamma ^\mu u + 2 \varepsilon _\mu q^\mu \bar{U} u/M)\right. \nonumber \\&\quad \left. +\, \frac{1}{\sqrt{2}} \tilde{a}^{\gamma q}_{1/2}( \varepsilon _\mu \bar{U} \gamma ^\mu \gamma ^5 u +2 \varepsilon _\mu q^\mu \bar{U} \gamma ^5 u/M) \right) ,\nonumber \\&\gamma ~\bar{q}\rightarrow \bar{X}:\ \delta ^b_a \left( \frac{1}{\sqrt{2}} a^{\gamma \bar{q}}_{1/2}( \varepsilon _\mu \bar{v} \gamma ^\mu V - 2 \varepsilon _\mu q^\mu \bar{v} V/M)\right. \nonumber \\&\quad \left. +\, \frac{1}{\sqrt{2}}\tilde{a}^{\gamma \bar{q}}_{1/2}( \varepsilon _\mu \bar{v} \gamma ^\mu \gamma ^5 V + 2 \varepsilon _\mu q^\mu \bar{v} \gamma ^5 V/M) \right) ,\nonumber \\&g~q\rightarrow X:\ T^{Ba}_b \left( \frac{1}{\sqrt{2}} a^{g q}_{1/2}( \varepsilon _\mu \bar{U} \gamma ^\mu u + 2 \varepsilon _\mu q^\mu \bar{U} u/M)\right. \nonumber \\&\quad \left. +\, \frac{1}{\sqrt{2}} \tilde{a}^{g q}_{1/2}( \varepsilon _\mu \bar{U} \gamma ^\mu \gamma ^5 u +2 \varepsilon _\mu q^\mu \bar{U} \gamma ^5 u/M) \right) ,\nonumber \\&g~\bar{q}\rightarrow \bar{X}:\ \tilde{T}^{Bb}_a \left( \frac{1}{\sqrt{2}} a^{g \bar{q}}_{1/2}( \varepsilon _\mu \bar{v} \gamma ^\mu V - 2 \varepsilon _\mu q^\mu \bar{v} V/M) \right. \nonumber \\&\quad \left. +\, \frac{1}{\sqrt{2}} \tilde{a}^{g \bar{q}}_{1/2}( \varepsilon _\mu \bar{v} \gamma ^\mu \gamma ^5 V + 2 \varepsilon _\mu q^\mu \bar{v} \gamma ^5 V/M) \right) . \end{aligned}$$

Spin 1:

$$\begin{aligned}&\gamma ~g\rightarrow X:\ \delta ^{AB}\left( a^{\gamma g}_{1} \left( \varepsilon ^{(\gamma )}_\mu {\varepsilon ^{(g)}}^\mu + 2 \varepsilon ^{(\gamma )}_\mu q^\mu \varepsilon ^{(g)}_\nu q^\nu /M^2\right) S^*_\rho q^\rho \right. \nonumber \\&\quad \left. +\, \tilde{a}^{\gamma g}_{1} S^*_\mu q^\mu \varepsilon ^{(\gamma )}_\nu \varepsilon ^{(g)}_\rho q_\lambda p_\sigma \epsilon ^{\nu \rho \lambda \sigma }/M^2 \right) ,\nonumber \\&g~g\rightarrow X:\ f^{ABB'}\left( a^{g g}_{1}\left( \varepsilon _\mu {\varepsilon '}^\mu + 2 \varepsilon _\mu q^\mu \varepsilon '_\nu q^\nu /M^2\right) S^*_\rho q^\rho \right. \nonumber \\&\quad \left. +\, \tilde{a}^{g g}_{1} S^*_\mu q^\mu \varepsilon _\nu \varepsilon '_\rho q_\lambda p_\sigma \epsilon ^{\nu \rho \lambda \sigma }/M^2\right) ,\nonumber \\&q~\bar{q}\rightarrow X:\ T^{Ab}_{b'}\left( a^{q \bar{q}}_{1} S_\mu ^* q^\mu \bar{v} u/M + \frac{1}{\sqrt{2}} b^{q \bar{q}}_{1} S_\mu ^* \bar{v} \gamma ^\mu u \right. \nonumber \\&\quad \left. +\, i \tilde{a}^{q \bar{q}}_{1} S_\mu ^* q^\mu \bar{v} \gamma ^5u /M + \frac{1}{\sqrt{2}} \tilde{b}^{q \bar{q}}_{1} S_\mu ^* \bar{v} \gamma ^\mu \gamma ^5u\right) . \end{aligned}$$

Spin $$\frac{3}{2}$$:

$$\begin{aligned}&\gamma ~q\rightarrow X:\ \delta ^a_b \left( \frac{\sqrt{3}}{2} a ^{\gamma q}_{3/2}( \varepsilon _\mu \bar{U}_\nu \gamma ^\mu u q^\nu /M + 2 \varepsilon _\mu q^\mu \bar{U}_\nu u q^\nu /M^2)\right. \nonumber \\&\quad \left. +\, b^{\gamma q}_{3/2}\left( \varepsilon ^\mu \bar{U}_\mu u - \frac{1}{2}\varepsilon _\mu \bar{U}_\nu \gamma ^\mu u q^\nu /M\right) \right. \nonumber \\&\quad \left. +\,\frac{\sqrt{3}}{2} \tilde{a}^{\gamma q}_{3/2}( \varepsilon _\mu \bar{U}_\nu \gamma ^\mu \gamma ^5 u q^\nu /M+ 2 \varepsilon _\mu q^\mu \bar{U}_\nu \gamma ^5 u q^\nu /M^2)\right. \nonumber \\&\quad \left. +\,i \tilde{b}^{\gamma q}_{3/2}\left( \varepsilon ^\mu \bar{U}_\mu \gamma ^5 u - \frac{1}{2}\varepsilon _\mu \bar{U}_\nu \gamma ^\mu \gamma ^5 u q^\nu /M\right) \right) ,\nonumber \\&\gamma ~\bar{q}\rightarrow \bar{X}:\ \delta ^b_a \left( \frac{\sqrt{3}}{2} a^{\gamma \bar{q}}_{3/2}( \varepsilon _\mu \bar{v} \gamma ^\mu V_\nu q^\nu /M - 2 \varepsilon _\mu q^\mu \bar{v} V_\nu q^\nu /M^2)\right. \nonumber \\&\quad \left. +\,b^{\gamma \bar{q}}_{3/2}\left( \varepsilon ^\mu \bar{v} V_\mu + \frac{1}{2}\varepsilon _\mu \bar{v} \gamma ^\mu V_\nu q^\nu /M\right) \right. \nonumber \\&\quad +\,\left. \frac{\sqrt{3}}{2} \tilde{a}^{\gamma \bar{q}}_{3/2}( \varepsilon _\mu \bar{v} \gamma ^\mu \gamma ^5 V_\nu q^\nu /M + 2 \varepsilon _\mu q^\mu \bar{v} \gamma ^5 V_\nu q^\nu /M^2)\right. \nonumber \\&\quad \left. +\,i \tilde{b}^{\gamma \bar{q}}_{3/2}\left( \varepsilon ^\mu \bar{v} \gamma ^5 V_\mu - \frac{1}{2}\varepsilon _\mu \bar{v} \gamma ^\mu \gamma ^5 V_\nu q^\nu /M\right) \right) ,\nonumber \\&g~q\rightarrow X:\ T^{Ba}_b \left( \frac{\sqrt{3}}{2} a ^{\gamma q}_{3/2}( \varepsilon _\mu \bar{U}_\nu \gamma ^\mu u q^\nu /M + 2 \varepsilon _\mu q^\mu \bar{U}_\nu u q^\nu /M^2)\right. \nonumber \\&\quad \left. +\, b^{\gamma q}_{3/2}\left( \varepsilon ^\mu \bar{U}_\mu u - \frac{1}{2}\varepsilon _\mu \bar{U}_\nu \gamma ^\mu u q^\nu /M\right) \right. \nonumber \\&\quad \left. +\,\frac{\sqrt{3}}{2} \tilde{a}^{\gamma q}_{3/2}( \varepsilon _\mu \bar{U}_\nu \gamma ^\mu \gamma ^5 u q^\nu /M + 2 \varepsilon _\mu q^\mu \bar{U}_\nu \gamma ^5 u q^\nu /M^2)\right. \nonumber \\&\quad \left. +\,i \tilde{b}^{\gamma q}_{3/2}\left( \varepsilon ^\mu \bar{U}_\mu \gamma ^5 u - \frac{1}{2}\varepsilon _\mu \bar{U}_\nu \gamma ^\mu \gamma ^5 u q^\nu /M\right) \right) ,\nonumber \\&g~\bar{q}\rightarrow \bar{X}:\ \tilde{T}^{Bb}_a \left( \frac{\sqrt{3}}{2} a^{g \bar{q}}_{3/2}( \varepsilon _\mu \bar{v} \gamma ^\mu V_\nu q^\nu /M - 2 \varepsilon _\mu q^\mu \bar{v} V_\nu q^\nu /M^2)\right. \nonumber \\&\quad \left. +\, b^{g \bar{q}}_{3/2}\left( \varepsilon ^\mu \bar{v} V_\mu + \frac{1}{2}\varepsilon _\mu \bar{v} \gamma ^\mu V_\nu q^\nu /M\right) \right. \nonumber \\&\quad \left. +\,\frac{\sqrt{3}}{2} \tilde{a}^{g \bar{q}}_{3/2}( \varepsilon _\mu \bar{v} \gamma ^\mu \gamma ^5 V_\nu q^\nu /M+ 2 \varepsilon _\mu q^\mu \bar{v} \gamma ^5 V_\nu q^\nu /M^2) \right. \nonumber \\&\quad \left. +\,i \tilde{b}^{g \bar{q}}_{3/2}\left( \varepsilon ^\mu \bar{v} \gamma ^5 V_\mu - \frac{1}{2}\varepsilon _\mu \bar{v} \gamma ^\mu \gamma ^5 V_\nu q^\nu /M\right) \right) . \end{aligned}$$

Spin 2:

$$\begin{aligned}&\gamma ~g\rightarrow X:\ \delta ^{AB}\left( \sqrt{\frac{3}{2}} a^{\gamma g}_2 \left( \varepsilon ^{(\gamma )}_\mu {\varepsilon ^{(g)}}^\mu + 2 \varepsilon ^{(\gamma )}_\mu q^\mu \varepsilon ^{(g)}_\nu q^\nu /M^2\right) \right. \nonumber \\&\quad \times \, S^*_{\rho \lambda } q^\rho q^\lambda /M \nonumber \\&\quad +\,\left. b^{\gamma g}_2 \left( M S^{\mu \nu *} \varepsilon ^{(\gamma )}_\mu \varepsilon ^{(g)}_\nu + \varepsilon ^{(g)}_\mu q^\mu S^{\nu \rho *} \varepsilon ^{(\gamma )}_\nu q_\rho /M\right. \right. \nonumber \\&\quad \left. \left. +\, \varepsilon ^{(\gamma )}_\mu q^\mu S^{\nu \rho *} \varepsilon ^{(g)}_\nu q_\rho /M - \frac{1}{2}{\varepsilon ^{(\gamma )}}^\mu \varepsilon ^{(g)}_\mu S^*_{\nu \rho } q^\nu q^\rho /M \right) \right. \nonumber \\&\quad +\,\sqrt{\frac{3}{2}} \tilde{a}^{\gamma g}_2 \varepsilon ^{(\gamma )}_\mu \varepsilon ^{(g)}_\nu S^*_{\rho \lambda } q^\rho q^\lambda \epsilon ^{\mu \nu \alpha \beta } q_\alpha p_\beta /M^3\nonumber \\&\quad \left. +\,\frac{1}{2}\tilde{b}^{\gamma g}_2\left( \varepsilon ^{(\gamma )}_\mu \varepsilon ^{(g)}_\nu S^*_{\gamma \rho } q^\rho \epsilon ^{\mu \nu \gamma \lambda } q_\lambda /M \right. \right. \nonumber \\&\quad \left. \left. +\,\varepsilon ^{(\gamma )}_\mu {\varepsilon ^{(g)}}^\rho S^*_{\nu \rho } \epsilon ^{\mu \nu \alpha \beta } q_\alpha p_\beta /M \right. \right. \nonumber \\&\quad \left. \left. +\, \varepsilon ^{(g)}_\mu {\varepsilon ^{(\gamma )}}^\rho S^*_{\nu \rho } \epsilon ^{\mu \nu \alpha \beta } q_\alpha p_\beta /M\right) \right) ,\nonumber \\&g~g\rightarrow X:\ d^{ABB'}\left( \sqrt{\frac{3}{2}} a^{g g}_2 \left( \varepsilon _\mu {\varepsilon '}^\mu + 2 \varepsilon _\mu q^\mu \varepsilon '_\nu q^\nu /M^2\right) \right. \nonumber \\&\quad \times \, S^*_{\rho \lambda } q^\rho q^\lambda /M \nonumber \\&\quad \left. +\,b^{g g}_2 \left( M S^{\mu \nu *} \varepsilon _\mu \varepsilon '_\nu + \varepsilon '_\mu q^\mu S^{\nu \rho *} \varepsilon _\nu q_\rho /M \right. \right. \nonumber \\&\quad \left. \left. +\, \varepsilon _\mu q^\mu S^{\nu \rho *} \varepsilon '_\nu q_\rho /M - \frac{1}{2}{\varepsilon }^\mu \varepsilon '_\mu S^*_{\nu \rho } q^\nu q^\rho /M \right) \right. \nonumber \\&\quad \left. +\,\sqrt{\frac{3}{2}} \tilde{a}^{g g}_2 \varepsilon _\mu \varepsilon '_\nu S^*_{\rho \lambda } q^\rho q^\lambda \epsilon ^{\mu \nu \alpha \beta } q_\alpha p_\beta /M^3\right) \nonumber \\&\quad +\, f^{ABB'} \left( \frac{1}{2}\tilde{b}^{g g}_2\left( \varepsilon _\mu \varepsilon '_\nu S^*_{\gamma \rho } q^\rho \epsilon ^{\mu \nu \gamma \lambda } q_\lambda /M \right. \right. \nonumber \\&\quad \left. \left. +\, \varepsilon _\mu {\varepsilon '}^\rho S^*_{\nu \rho } \epsilon ^{\mu \nu \alpha \beta } q_\alpha p_\beta /M\right. \right. \nonumber \\&\quad \left. \left. +\, \varepsilon '_\mu {\varepsilon }^\rho S^*_{\nu \rho } \epsilon ^{\mu \nu \alpha \beta } q_\alpha p_\beta /M\right) \right) ,\nonumber \\&q~\bar{q}\rightarrow X:\ T^{Ab}_{b'}\left( \sqrt{\frac{3}{2}} a^{q \bar{q}}_2 S_{\mu \nu }^* q^\mu q^\nu \bar{v} u /M^2\right. \nonumber \\&\quad +\, b^{q \bar{q}}_2 S_{\mu \nu }^* q^\nu \bar{v} \gamma ^\mu u/M +\,\sqrt{\frac{3}{2}} i \tilde{a}^{q \bar{q}}_2 S_{\mu \nu }^* q^\mu q^\nu \bar{v} \gamma ^5u/M^2 \nonumber \\&\quad \left. +\, \tilde{b}^{q \bar{q}}_2 S_{\mu \nu }^* q^\nu \bar{v} \gamma ^\mu \gamma ^5u/M\phantom {\sqrt{\frac{3}{2}}}\right) . \end{aligned}$$

The amplitudes involving two incoming gluons respect Bose symmetry under the exchange: $B \leftrightarrow B'$, $\varepsilon \leftrightarrow \varepsilon '$, $q \leftrightarrow -q$.

It is then straightforward to write down the non-zero production amplitudes $_{\mathrm {out}}\langle X, m| \mathcal {P}, \lambda ; \mathcal {P}', \lambda '\rangle _{\mathrm {in}}$ for the process where parton $\mathcal {P}$ with helicity $\lambda$ coming along the positive $\hat{z}$ axis ($\theta = 0$) and parton $\mathcal {P}'$ with helicity $\lambda '$ coming along the negative $\hat{z}$ axis ($\theta = \pi$) create a resonance of spin *j* and spin projection along $\hat{z}$ equal to $m = \lambda - \lambda '$. As already mentioned, the coefficients *a* refer to the pairs of lowest helicity (RR or LL) while *b* refers to RL or LR, if present.

The CPT theorem,

1 $$\begin{aligned}&_{\mathrm {out}}\langle \mathcal {P}, \lambda ; \mathcal {P}', \lambda ' | X, m \rangle _{\mathrm {in}}\nonumber \\&\quad = (-)^{j-m}~_{\mathrm {out}}\langle \bar{X}, -m| \bar{\mathcal {P}}, -\lambda ; \bar{\mathcal {P}'}, -\lambda '\rangle _{\mathrm {in}}, \end{aligned}$$

allows then one to write down the $1\rightarrow 2$ amplitudes for the decay of the resonance *X* in its rest frame into one parton $\mathcal {P}$ and a photon.

If one assumes the hermiticity of the effective interaction giving rise to the couplings (e.g. when the interaction arises by integrating out heavy mediators, as also discussed in [37]), one can assume $_{\mathrm {out}}\langle X, m| \mathcal {P}, \lambda ; \mathcal {P}', \lambda '\rangle _{\mathrm {in}} = \; _{\mathrm {out}}\langle \mathcal {P}, \lambda ; \mathcal {P}', \lambda ' |X, m \rangle ^*_{\mathrm {in}}$. The above assumption, combined with the CPT theorem, forces the coefficients of the integer-spin amplitudes to be real and those of the half-integer ones to be conjugate of each other. In the simulations of Sect. 3 we will always assume this to be the case.

### The jet+*Z* boson case

For the decay into a jet and a *Z* boson we need to generalise some of the amplitudes in the previous section to include a massive vector boson. The notation is as before, but now $p_1^\mu$ is the momentum of the *Z* boson, with $p_1^2 = m_Z^2$. The other parton is still massless and $q=p_1-p_2$ and $p=p_1+p_2$ now obey $q^2 = 2 m_Z^2 - M^2$, $p^2 = M^2$ and $p\cdot q=m_Z^2$. The energy of the massless parton in the centre-of-mass frame is now $(M^2-m_Z^2)/2M$, so the on-shell normalisation of the quark wave-function has changed. All remaining polarisations are the same and, for the *Z*, we have an additional longitudinal polarisation. In order not to confuse the gluon and the *Z* polarisation, we refer to the gluon as $\varepsilon ^\mu$ and denote the *Z* polarisations by $\zeta ^\mu$.

We write down the production amplitudes for the process $Z\, \mathcal {P}\rightarrow X$. Of course, in this case one is really only interested in the conjugate process $X\rightarrow \,Z \mathcal {P}$, but we stick with this notation for ease of comparison with the previous formulas. The coefficients are chosen in such a way that for $m_Z\rightarrow 0$ these amplitudes reduce to the previous one with a photon.

Spin 0:

$$\begin{aligned}&Z~g\rightarrow X:\ \delta ^{AB}\left( a^{Z g}_0 \left( (M^2-m_Z^2)\zeta _\mu {\varepsilon }^\mu + 2 \zeta _\mu q^\mu \varepsilon _\nu q^\nu \right) /M\right. \nonumber \\&\quad \left. +\,\tilde{a}^{Z g}_0 \zeta _\mu \varepsilon _\nu q_\lambda p_\rho \epsilon ^{\mu \nu \lambda \rho }/M\right) . \end{aligned}$$

Spin $$\frac{1}{2}$$:

$$\begin{aligned}&Z~q\rightarrow X:\ \delta ^a_b \left( \frac{1}{\sqrt{2}}a^{Z q}_{1/2}( (M^2-m_Z^2)\zeta _\mu \bar{U} \gamma ^\mu u \right. \nonumber \\&\quad +\, 2 M \zeta _\mu q^\mu \bar{U} u)/M^2 + 2\, c^{Z q}_{1/2}\, m_Z^2 \zeta ^\mu q_\mu \bar{U} u /M^3 \nonumber \\&\quad +\, \frac{1}{\sqrt{2}} \tilde{a}^{Z q}_{1/2}( (M^2-m_Z^2) \zeta _\mu \bar{U} \gamma ^\mu \gamma ^5 u + 2 M \zeta _\mu q^\mu \bar{U} \gamma ^5 u)/M^2\nonumber \\&\quad \left. +\,2 \, \tilde{c}^{Z q}_{1/2}\, m_Z^2 \zeta ^\mu q_\mu \bar{U} \gamma ^5 u / M^3 \phantom {\frac{1}{\sqrt{2}}}\right) ,\nonumber \\&Z~\bar{q}\rightarrow \bar{X}:\ \delta ^b_a \left( \frac{1}{\sqrt{2}} a^{Z \bar{q}}_{1/2}( (M^2- m_Z^2)\zeta _\mu \bar{v} \gamma ^\mu V \right. \nonumber \\&\quad -\, 2 M \zeta _\mu q^\mu \bar{v} V)/M^2 +2\, c^{Z \bar{q}}_{1/2}\, m_Z^2 \zeta ^\mu q_\mu \bar{v} V /M^3 \nonumber \\&\quad +\, \frac{1}{\sqrt{2}} \tilde{a}^{Z \bar{q}}_{1/2}( (M^2 - m_Z^2)\zeta _\mu \bar{v} \gamma ^\mu \gamma ^5 V + 2 M \zeta _\mu q^\mu \bar{v} \gamma ^5 V)/M^2 \nonumber \\&\quad \left. +\, 2 \, \tilde{c}^{Z \bar{q}}_{1/2}\, m_Z^2 \zeta ^\mu q_\mu \bar{v} \gamma ^5 V /M^3 \phantom {\frac{1}{\sqrt{2}}}\right) . \end{aligned}$$

Spin 1:

$$\begin{aligned}&Z~g\rightarrow X:\ \delta ^{AB}\bigg (a^{Z g}_{1} \left( (M^2-m_Z^2)\zeta _\mu {\varepsilon }^\mu + 2 \zeta _\mu q^\mu \varepsilon _\nu q^\nu \right) \nonumber \\&\quad \times \, S^*_\rho q^\rho /M^2 \nonumber \\&\quad +\,2\, c^{Z g}_{1}\, m_Z^2\left( (M^2-m_Z^2) \varepsilon ^\mu S^*_\mu \zeta ^\nu q_\nu + \zeta ^\mu q_\mu \varepsilon ^\nu q_\nu S^{*\rho } q_\rho \right) /M^4 \nonumber \\&\quad +\, \tilde{a}^{Z g}_{1} S^*_\mu q^\mu \zeta _\nu \varepsilon _\rho q_\lambda p_\sigma \epsilon ^{\nu \rho \lambda \sigma }/M^2 \nonumber \\&\quad +\, 2\, \tilde{c}^{Z g}_{1}\, m_Z^2 \zeta ^\mu q_\mu \varepsilon _\nu S^*_\rho q_\lambda p_\sigma \epsilon ^{\nu \rho \lambda \sigma }/M^4 \bigg ). \end{aligned}$$

Spin $$\frac{3}{2}$$:

$$\begin{aligned}&Z~q\rightarrow X:\ \delta ^a_b \left( \frac{\sqrt{3}}{2} a ^{Z q}_{3/2}( (M^2 - m_Z^2)\zeta _\mu \bar{U}_\nu \gamma ^\mu u q^\nu \right. \nonumber \\&\quad +\, 2 M \zeta _\mu q^\mu \bar{U}_\nu u q^\nu )/M^3\nonumber \\&\quad +\, b^{Z q}_{3/2}( (M^2 -m_Z^2)^2 \zeta ^\mu \bar{U}_\mu u - \frac{1}{2}M (M^2-m_Z^2)\nonumber \\&\quad \zeta _\mu \bar{U}_\nu \gamma ^\mu u q^\nu + m_Z^2 \zeta ^\mu q_\mu q^\nu \bar{U}_\nu u )/M^4 \nonumber \\&\quad +\, \sqrt{6}\, c^{Z q}_{3/2}\, m_Z^2 \zeta ^\mu q_\mu q^\nu \bar{U}_\nu u/M^4 \nonumber \\&\quad +\, \frac{\sqrt{3}}{2} \tilde{a} ^{Z q}_{3/2}( (M^2 - m_Z^2)\zeta _\mu \bar{U}_\nu \gamma ^\mu \gamma ^5 u q^\nu \nonumber \\&\quad +\, 2 M \zeta _\mu q^\mu \bar{U}_\nu \gamma ^5 u q^\nu )/M^3\nonumber \\&\quad +\,i\, \tilde{b}^{Z q}_{3/2}\left( (M^2 -m_Z^2)^2 \zeta ^\mu \bar{U}_\mu \gamma ^5 u \phantom {\frac{1}{\sqrt{2}}}\right. \nonumber \\&\quad -\, \frac{1}{2}M (M^2-m_Z^2)\zeta _\mu \bar{U}_\nu \gamma ^\mu \gamma ^5 u q^\nu \nonumber \\&\quad \left. +\, m_Z^2 \zeta ^\mu q_\mu q^\nu \bar{U}_\nu \gamma ^5 u \phantom {\frac{1}{\sqrt{2}}}\right) /M^4 \nonumber \\&\quad \left. +\,\sqrt{6} \, \tilde{c}^{Z q}_{3/2}\, m_Z^2 \zeta ^\mu q_\mu q^\nu \bar{U}_\nu \gamma ^5 u/M^4\phantom {\frac{1}{\sqrt{2}}}\right) ,\nonumber \\&Z~\bar{q}\rightarrow \bar{X}:\ \delta ^b_a \left( \frac{\sqrt{3}}{2} a^{Z \bar{q}}_{3/2}( (M^2 - m_Z^2) \zeta _\mu \bar{v} \gamma ^\mu V_\nu q^\nu \right. \nonumber \\&\quad -\, 2 M\zeta _\mu q^\mu \bar{v} V_\nu q^\nu )/M^3\nonumber \\&\quad +\,b^{Z \bar{q}}_{3/2}((M^2 -m_Z^2)^2\zeta ^\mu \bar{v} V_\mu + \frac{1}{2}M (M^2-m_Z^2)\zeta _\mu \bar{v} \gamma ^\mu V_\nu q^\nu \nonumber \\&\quad +\, m_Z^2 \zeta ^\mu q_\mu \bar{v} V_\nu q^\nu )/M^4 \nonumber \\&\quad +\, \sqrt{6}\, c^{Z \bar{q}}_{3/2}\, m_Z^2 \zeta ^\mu q_\mu \bar{v} V_\nu q^\nu /M^4 \nonumber \\&\quad +\,\frac{\sqrt{3}}{2} \tilde{a}^{Z \bar{q}}_{3/2}( (M^2 - m_Z^2) \zeta _\mu \bar{v} \gamma ^\mu \gamma ^5 V_\nu q^\nu \nonumber \\&\quad +\, 2 M \zeta _\mu q^\mu \bar{v} \gamma ^5 V_\nu q^\nu )/M^3\nonumber \\&\quad +\,i\, \tilde{b}^{Z \bar{q}}_{3/2}\left( (M^2 -m_Z^2)^2\zeta ^\mu \bar{v} \gamma ^5 V_\mu \phantom {\frac{1}{\sqrt{2}}}\right. \nonumber \\&\quad -\, \frac{1}{2}M (M^2-m_Z^2)\zeta _\mu \bar{v} \gamma ^\mu \gamma ^5 V_\nu q^\nu \nonumber \\&\quad \left. +\, m_Z^2 \zeta ^\mu q_\mu \bar{v} \gamma ^5 V_\nu q^\nu \phantom {\frac{1}{\sqrt{2}}}\right) /M^4 \nonumber \\&\quad \left. +\, \sqrt{6} \, \tilde{c}^{Z \bar{q}}_{3/2}\, m_Z^2 \zeta ^\mu q_\mu \bar{v} \gamma ^5 V_\nu q^\nu /M^4 \phantom {\frac{1}{\sqrt{2}}}\right) . \end{aligned}$$

Spin 2:

$$\begin{aligned}&Z~g\rightarrow X:\ \delta ^{AB}\left( \sqrt{\frac{3}{2}} a^{Z g}_{2} ((M^2 - m_Z^2)\zeta _\mu {\varepsilon }^\mu \right. \nonumber \\&\quad +\, 2 \zeta _\mu q^\mu \varepsilon _\nu q^\nu )S^*_{\rho \lambda } q^\rho q^\lambda /M^3 \nonumber \\&\quad +\, b^{Z g}_{2} \left( (M^2 - m_Z^2)^3 S^{\mu \nu *} \zeta _\mu \varepsilon _\nu \phantom {\frac{1}{\sqrt{2}}}\right. \nonumber \\&\quad +\,(M^2 - m_Z^2)^2 \varepsilon _\mu q^\mu S^{\nu \rho *} \zeta _\nu q_\rho \nonumber \\&\quad +\,(M^4 - m_Z^4)\zeta _\mu q^\mu S^{\nu \rho *} \varepsilon _\nu q_\rho \nonumber \\&\quad -\,\frac{1}{2}M^2 (M^2 - m_Z^2){\zeta }^\mu \varepsilon _\mu S^*_{\nu \rho } q^\nu q^\rho \nonumber \\&\quad \left. +\, m_Z^2{\zeta }^\mu q_\mu \varepsilon ^\nu q_\nu S^*_{\rho \lambda } q^\rho q^\lambda \phantom {\frac{1}{\sqrt{2}}}\right) /M^5 \nonumber \\&\quad +\,2 \sqrt{2}\, c^{Z g}_{2} \, m_Z^2((M^2-m_Z^2) S^{\mu \nu *} \varepsilon _\mu q_\nu \zeta ^\rho q_\rho \nonumber \\&\quad +\, S^{\mu \nu *} q_\mu q_\nu \varepsilon ^\lambda q_\lambda \zeta ^\rho q_\rho )/M^5\nonumber \\&\quad +\, \sqrt{\frac{3}{2}} \tilde{a}^{Z g}_{2} \zeta _\mu \varepsilon _\nu S^*_{\rho \lambda } q^\rho q^\lambda \epsilon ^{\mu \nu \alpha \beta } q_\alpha p_\beta /M^3 \nonumber \\&\quad +\,\tilde{b}^{Z g}_{2} \left( (M^2-m_Z^2)^2 \epsilon ^{\mu \nu \gamma \lambda } S^*_{\mu \rho }\zeta ^\rho \varepsilon _\nu q_\gamma p_\lambda \phantom {\frac{1}{\sqrt{2}}}\right. \nonumber \\&\quad -\,\frac{1}{2}M^2 S^*_{\mu \nu }q^\mu q^\nu \epsilon ^{\rho \lambda \alpha \beta } \zeta _\rho \varepsilon _\lambda q_\alpha p_\beta \nonumber \\&\quad \left. -\,(M^2+m_Z^2) \zeta ^\mu q_\mu \epsilon ^{\rho \lambda \alpha \beta } \varepsilon _\rho S^*_{\lambda \sigma } q^\sigma q_\alpha p_\beta \phantom {\frac{1}{\sqrt{2}}}\right) /M^5 \nonumber \\&\quad \left. +\,2\sqrt{2}\, \tilde{c}^{Z g}_{2}\, m_Z^2 (\zeta ^\mu q_\mu \epsilon ^{\nu \rho \lambda \sigma } \varepsilon _\nu S^*_{\rho \gamma } q^\gamma q_\lambda p_\sigma )/M^5 \phantom {\frac{1}{\sqrt{2}}}\right) . \end{aligned}$$

Although the purpose of this work is to be as model independent as possible, it is worth commenting on possible scenarios in which such couplings could arise. Historically, bosonic resonances of this type were considered in the context of technicolour models [4] while fermionic resonances arose considering models of quark compositeness [20]. These particles can be pair produced with ordinary QCD strength and this puts model-independent bounds on the low mass region of the spectrum, typically below 1 TeV. The single production modes, via gluon–gluon or quark–gluon fusion, of interest to us have a higher mass reach but are more model dependent.

As far as bosonic resonances are concerned, while the original motivation from technicolour has greatly diminished due to the phenomenological difficulties of these models, recently there has been interest in the search for coloured pseudo-Nambu–Goldstone bosons that arise in more recent models of partial compositeness [7, 16]. The production of such objects occurs via the anomaly and the cross section scales like $(K/f)^2$, where *K* is the anomaly coefficient and *f* the decay constant. For $K/f =1/\text{ TeV }$ the production cross section ranges from 0.1 pb for $M=500$ GeV to 0.1 fb for $M=3$ TeV at $\sqrt{s}= 13$ TeV. Here the bounds are still attractive for LHC searches, allowing, for some models, masses around 1 TeV for values of the decay constant of 1 TeV.

As far fermionic resonances are concerned, the recent CMS search [35] using the model [22] sets a bound of 5.5 TeV on first and second family excited quarks for amplitude coefficient $a_{1/2}^{gq}$ $a_{1/2}^{\gamma q}$ of order $g_s$, *e*, respectively, and 1.8 TeV for excited *b*-quarks. The production cross section times branching ratio into jet-$\gamma$ are about 0.2 and 7 fb at the upper limit of the excluded mass range. For amplitude coefficients reduced by a factor 10, the mass reach is below 2 TeV for light quarks while the search lacks sufficient sensitivity to set a bound for the *b*-quark.

## Elements of hadron collider phenomenology

To gain a quantitative understanding of the phenomenology that we can expect at the LHC, we first consider the most motivated cases of scalars, spin $\frac{1}{2}$ fermions and vector resonances. We later shall comment on the qualitative differences compared to the higher spin modes for representative examples, thus generalising the analysis of [32–35] to all allowed coupling structures in the light of CPT and Lorentz invariance.

We have implemented the couplings of the previous section into the MadEvent [38, 39] event generator with purpose-built Helas routines [40], and have checked these against implementations derived from the FeynRules [41] and Ufo [42] toolkits. As already mentioned, we only consider flavour symmetric cases and treat all quarks in the four-flavour scheme (and hence the involved pdfs) on an equal footing ($q=u,d,c,s$ in the following). We interface the generated parton-level events with Herwig++ [43, 44] for showering and hadronisation, and choose as benchmark $M=500~{\text {GeV}}$ for demonstration purposes. We will point out the influence of the mass scale *M* on our analysis below, where we will also comment on detector resolution effects. Throughout, we focus on 13 TeV collisions.

Due to the very character of the above amplitudes being decompositions of physical scattering amplitudes in the narrow width approximation, we choose a small reference width $\Gamma _X/M=10^{-4}$. In this work we will not discuss the extraction of the coupling sizes comprehensively, but we note that our analysis of the signal events is entirely insensitive to the exact choice as long as $\Gamma _X/M\ll 1$. Since the amplitudes of Sect. 2 are not valid in the off-shell regime of any involved legs, we can expect measurements of the CP character of the resonance along the lines of [45–51], when relevant, to be significantly limited already at this stage. The non-validity of the above amplitudes for off-shell momenta also does not allow us to perform multi-jet matching for the signal.

We have simulated the contributing backgrounds in an identical way and specifically focus on QCD-induced $\gamma /Z$+jet production which are the largest contributing backgrounds in the SM. The LHC experiments typically estimate these with data-driven methods (see e.g. [32]) and this part of our analysis solely serves as a numerical guide to highlight the potential sensitivity for our benchmark. To allow us to compare signal and background on an equal footing, we do not include jet-matching effects for the backgrounds.

Since the resonances we study in this work are motivated from general amplitude Lorentz structures, and since these carry colour charges, such a discovery can be established not only $\gamma /Z+\hbox {jet}$ production, but also in multi-jet final states. Analyses of the latter have been carried out in two jet [52, 53] and in four jet final states [54, 55]. A discovery will crucially depend on the sizes of the coefficients quoted in Sects. 2.1 and 2.2, as these can be chosen to avoid discovery in the multi-jet channels.

The LHC already performs bump hunts for resonances in the $\gamma +\hbox {jet}$ channel as detailed in [32–35]. These are performed by a data-driven estimate of the sensitive invariant mass distribution with the aim to reveal excesses in model-independent approach. If such a search is successful, the question of the precise coupling structure of the new discovery arises. The *Z* boson takes a special role in this case due to gauge invariance (however, at a much smaller rate due to leptonic branching ratios which deliver the cleanest signatures for subsequent analyses). The imminent spectroscopy programme after such a discovery will then need to be informed by a range of searches, in particular because the exclusive rate of decays into the final states we discuss in this work will be influenced by the coefficients of the di-jet channels. For the purpose of this work we will assume a discovery in the jet+$\gamma$ channel at 300/fb (this might be accompanied by a similar observation in the multi-jet channels) and we outline a spectroscopy follow-up programme in the jet+$\gamma /Z$ channels. We will come back to the importance of multi-jet final states at the end of this section.

Different spin expectations can be discriminated by characteristic angular distributions [18, 56–61] (see also [24, 29, 62]). The angles that are sensitive to the spin structure of the interactions which are relevant to our final states are

2 $$\begin{aligned}&\cos \theta _h = { {\vec {p}}_{\ell ^-} \cdot {\vec {p}}_{X} \over \sqrt{ {\vec {p}}^{\,2}_{\ell ^-}\, {\vec {p}}^{\,2}_{X} }} \bigg |_{Z}, \end{aligned}$$

3 $$\begin{aligned}&\cos \phi = { (\hat{e}_z\times \hat{e}_{v})\cdot ({\vec {p}}_{\ell ^-} \times {\vec {p}}_{\ell ^+}) \over \sqrt{( {\vec {p}}_{\ell ^-} \times {\vec {p}}_{\ell ^+} )^2} } \bigg |_X , \end{aligned}$$

4 $$\begin{aligned}&\cos \theta ^*= { {\vec {p}}_{V} \cdot {\hat{e}_{z}} \over \sqrt{ {\vec {p}}^{\,2}_{V} }} \bigg |_{X}. \end{aligned}$$

**Fig. 1:**
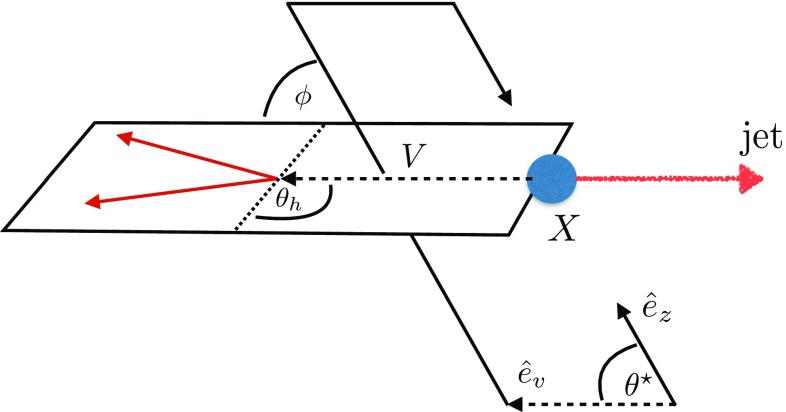
Illustration of the angles sensitive to the spin and polarisation information discussed in this work. $\hat{e}_z$ denotes the normalised direction of the proton–proton beam axis, $\hat{e}_v$ denotes the direction of *Z* or $\gamma$ boson in the particle *X* rest frame. In the case of $Z+\hbox {jet}$ production the *Z* boson lepton decay angles include important information: $\theta _h$ denotes the angle of the negatively charged lepton against the resonance *X* in the *Z* boson rest frame. $\phi$ denotes the angle between the production and decay planes

The subscripts *X*, *Z* refer to the rest frames in which these angles are defined. The momenta are defined from the decay products, i.e.

5 $$\begin{aligned} \vec {p}_X=\vec {p}_{\gamma /Z} + \vec {p}_j \end{aligned}$$

with

6 $$\begin{aligned} \vec {p}_Z=\vec {p}_{\ell ^-} + \vec {p}_{\ell ^+} \end{aligned}$$

in the case of $\hbox {jet}+Z$ production. Note that the helicity angle $\theta _h$ and the azimuthal angle $\phi$ are not observable for decays $X\rightarrow \gamma j$, hence limiting the available range of sensitive observables to $\theta ^*$. We have introduced $\phi$ for completeness but we find that it contains no discriminative power for the scenarios we study in this work, and we will not further consider this angle in the following (Fig. 1).

**Table 1:** Central value cross sections for the $\gamma$+jet scenarios discussed in Sect. 3 for coupling values of $10^{-1}$. The results for P/CP-violating parameters are identical

Scenario	$\sigma (M=500~\text {GeV})$ [pb]	$\Gamma _X$ [GeV]	BR$(X\rightarrow \gamma j)$
${a}^{gg}_0 \otimes {a}^{\gamma g}_0$	83.58	0.36	0.55
${a}^{q\bar{q}}_0\otimes {a}^{\gamma g}_0$	48.83	0.60	0.33
$a^{gq}_{1/2} \otimes {a}^{\gamma q}_{1/ 2}$	82.54	0.93	0.43
$a^{gg}_1 \otimes {a}^{\gamma g}_{1}$	127.2	0.16	0.40
$a^{q \bar{q} }_{1} \otimes {a}^{\gamma g}_{1}$	22.22	0.20	0.33
$b^{q \bar{q} }_{1} \otimes {a}^{\gamma g}_{1}$	25.45	0.20	0.33
$a^{gq}_{3/2} \otimes a^{\gamma q}_{3/2}$	214.9	0.46	0.43
$a^{gg}_{2} \otimes a^{\gamma g}_2$	68.71	0.07	0.55

**Table 2:** Central value cross sections for the *Z*+jet scenarios discussed in Sect. 3 for coupling values of $10^{-1}$. The results for P/CP-violating parameters are identical

Scenario	$\sigma (M=500~\text {GeV})$	$\Gamma _X$ [GeV]	BR$(X\rightarrow Z j)$
${a}^{gg}_0 \otimes {a}^{Z g}_0$	66.00	0.36	0.54
$a^{gq}_{1/2} \otimes {a}^{Z q}_{1/ 2}$	314.0	0.92	0.43
$a^{gg}_1 \otimes {a}^{Z g}_{1}$	105.9	0.17	0.40
$a^{gg}_1 \otimes {c}^{Z g}_{1}$	30.70	0.11	0.08
$a^{gq}_{3/2} \otimes a^{Z q}_{3/2}$	74.03	0.46	0.42
$a^{gg}_{2} \otimes a^{Z g}_2$	53.97	0.07	0.55

The discriminating power of these angles (we will discuss the contributing backgrounds further below) lies in the fact that the boosts into respective rest frames remove some kinematic dependence on the final states and the mass of *X* in particular. For the jet-$\gamma$ case, in the lab frame, the scattering is fully described through a combination of transverse momentum $p_T$ and pseudorapidity $\eta$ of the photon.

While removing this energy dependence has the benefit of projecting out the helicity decomposition of the interactions of Sect. 2 (sculpted to some extent by finite detector coverage), the distribution of the signal events according to energy-sensitive observables such as the transverse photon momentum can provide evidence of the dominant production mechanism via pdf effects. Another avenue we will discuss in the following is the prospects of quark–gluon tagging [63–65], which can in principle further discriminate the *X* decay phenomenology, thus providing important information in discriminating the different spin hypotheses. This strategy is particularly motivated, as the threshold induced by the expected large mass of *X* helps to choose working points that are particularly attractive to quark–gluon tagging [66].

Our event selection is performed fairly inclusively only reflecting the basic trigger thresholds for the signal events to be recorded. Representative generator level cross sections for the coupling combinations that we study in this paper are tabulated in Tables 1 and 2.

**Fig. 2:**
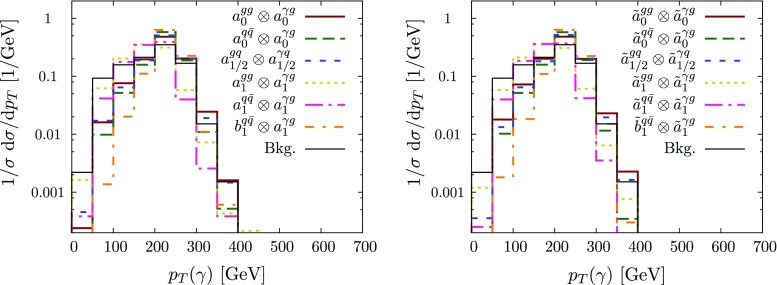
Normalised transverse momentum distribution for jet+$\gamma$ production, focussing on the different spin *j* (here specifically $j\le 1$) and types of coupling detailed in Sect. 2. The lower index denotes the spin *j*. The symbol $\otimes$ separates the coefficients involved in the production and decay of the resonance. The resonance mass *M* is set at 500 GeV, merging or detector effects are not included here

**Fig. 3:**
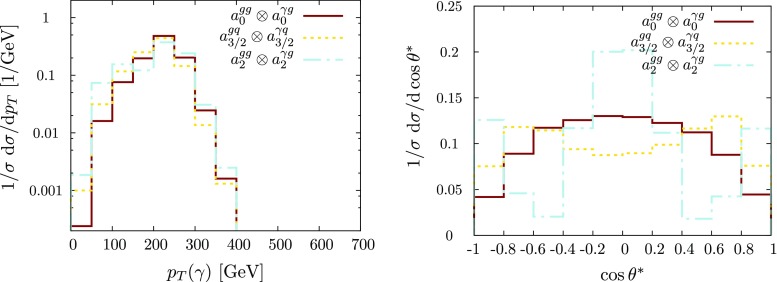
Normalised transverse momentum and $\cos \theta ^*$ distributions for jet+$\gamma$ production, focussing on representative spin 3 / 2 and spin 2 couplings of Sect. 2. (Spin 0 included for comparison.) See caption of Fig. 2 for further details. No merging or detector resolution effects are included

For the case of photon-associated jet production we require an isolated photon (defined as isolated if the hadronic energy deposit in an area $R<0.3$ around the photon candidate is less than 5% of the photon’s transverse momentum) with

7 $$\begin{aligned} p_{T,\gamma } >30~\text {GeV},\quad \hbox {and}\quad |\eta _\gamma | < 2.33, \end{aligned}$$

to guarantee the event to be triggered [67]. In the case of final state leptons, we require two isolated leptons (hadronic energy deposit less than 10% of the lepton candidate’s transverse momentum in $R<0.3$) with opposite charge and

8 $$\begin{aligned} p_{T,\ell } >30~\text {GeV},\quad \hbox {and}\quad |\eta _\ell | < 2.5. \end{aligned}$$

Again these criteria reflect the standard trigger thresholds [67]. On top of these thresholds we require the leptons to be compatible with the *Z* pole mass,

9 $$\begin{aligned} |m_{\ell ^+\ell ^-}-m_Z|<5~\text {GeV}, \end{aligned}$$

in the leptonic final state case.

The jets are clustered with the anti-kT algorithm [68] with resolution parameter $D=0.4$ using FastJet [69] and we define jets from the thresholds by

10 $$\begin{aligned} p_{T,j} >30~\text {GeV},\quad \hbox {and}\quad |\eta _j| < 4.5. \end{aligned}$$

We require the leading jet in $p_T$ to be inside $|\eta _{j_1}| < 2.33$ in a back-to-back configuration with the reconstructed photon or *Z* boson ($p_Z=p_{\ell ^-} +p_{\ell ^+}$) in the azimuthal angle–pseudorapidity plane $R(j,Z/\gamma )>2.5$. Finally we require consistency of the reconstructed resonance with our mass hypothesis within 50 GeV,

11 $$\begin{aligned} |m_{j\gamma /Z}-M| < 25~\text {GeV}. \end{aligned}$$

This latter criterion, while not relevant for our signal distributions, is crucial for comparison of the signal with the expected background. For comparison, ATLAS searches are sensitive to width/mass ratios of 2% in Ref. [32], which is well covered by our representative invariant mass window cut in our signal-like selection, where our approximations can be trusted.

**Fig. 4:**
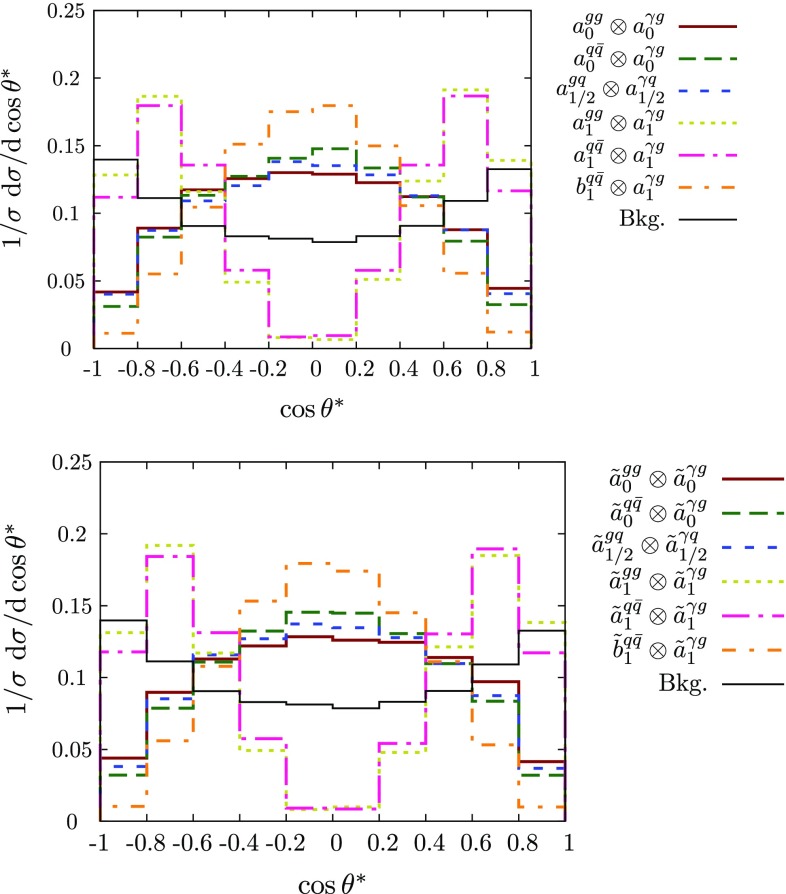
Normalised $\cos \theta ^*$ distribution for jet+$\gamma$ production for various spin and couplings (not considering detector resolution or jet-merging effects). The distributions do not allow one to discriminate between the P/CP properties of the interactions

For the jet$+\gamma$ case, where we essentially only have a single angle at our disposal to discriminate the various hypotheses, we can already identify the qualitative overall behaviour of the final state. The $p_T$ distribution of the photon (Figs. 2 and 3), while giving some indication of the dominant partonic subprocess through the parton distribution functions as well as spin and coupling character, is largely dominated by the threshold of the particle *X*, whose mass gets equally distributed into transverse momentum for central production. The differential measurement of $\theta ^*$ (Figs. 3 and 4), on the other hand, allows us to formidably discriminate between different spin hypotheses. Some of the remaining qualitative degeneracies can be lifted (see below).^1^

The background distribution is fundamentally different from the signal distribution. As alluded to above, none of the observables of the hard $2\rightarrow 2$ scattering reflects the P/CP character of the couplings and more involved processes that access off-shell information need to be considered (see Fig. 4).

**Fig. 5:**
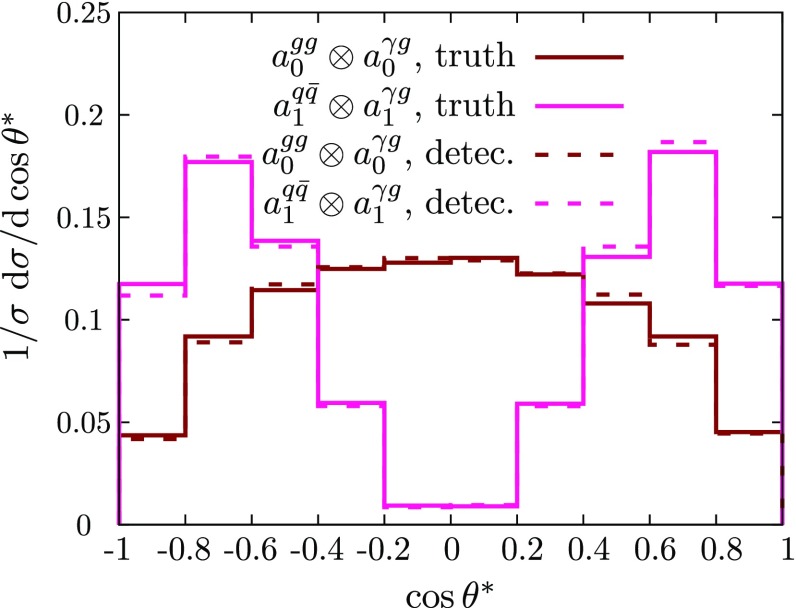
Impact of energy mis-measurements on the $\cos \theta ^\star$ distribution for our benchmark scenario of $M=500$ GeV for two representative spin and coupling hypotheses

**Fig. 6:**
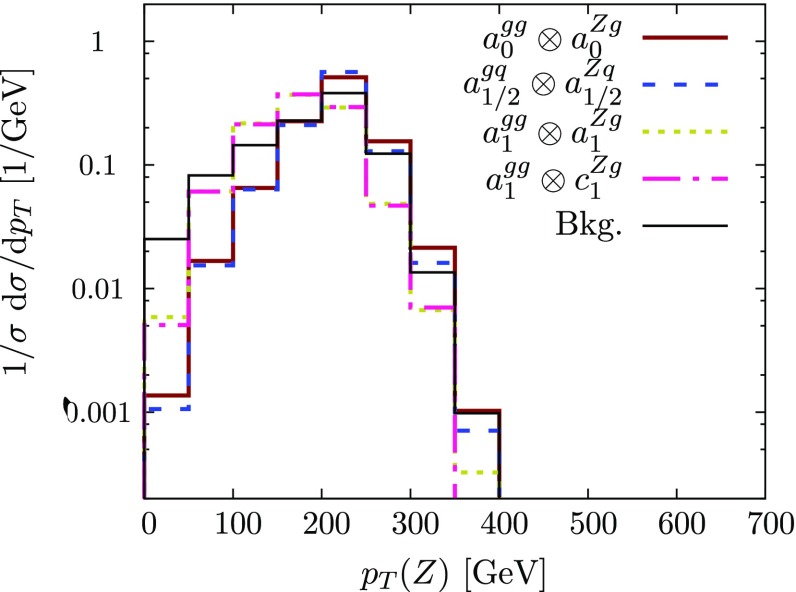
Normalised transverse momentum distribution for jet+*Z* production, focussing on representative spin 0, 1 / 2, 1 and coupling properties of Sect. 2 (no jet-merging and detector resolution effects). See caption of Fig. 2 for further details on the notation

**Fig. 7:**
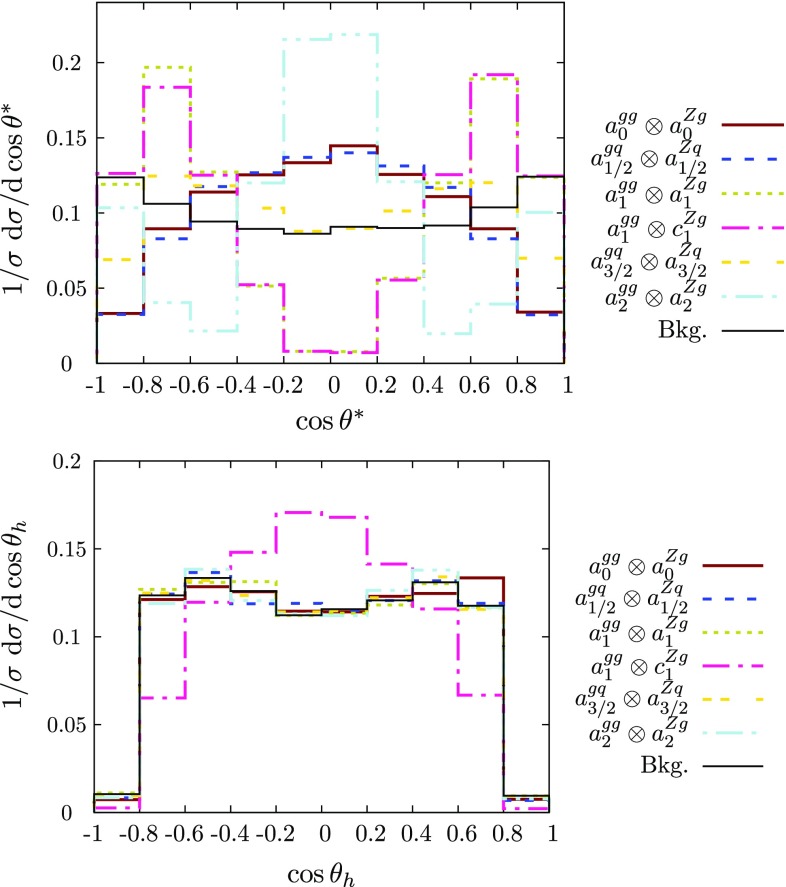
Normalised $\cos \theta ^*$ and helicity angle distributions for jet+*Z* production for the different spin and coupling properties as detailed in Sect. 2 (no jet-merging and detector resolution effects are included). We include the expected background distributions. See caption of Fig. 2 for further details on the notation

All results in Figs. 2, 3 and 4 do not include the expected effects of energy mis-measurements. As the sensitivity in these angle depends on the correct reconstruction of the *X* rest frame, detector resolution effects can in principle limit the sensitivity. In practice, when the mass scales are large, the expected calibration of photons and jets has shown to be under extremely good control and is likely to improve even further in the future [70]. However, to provide a more quantitative understanding of how detector effects modify the angular distributions we show a comparison of $\cos \theta ^\star$ including detector effects in Fig. 5 (we have adopted the energy parameterisation of Delphes3 [71]).

This phenomenological situation remains largely unchanged when considering the *Z* boson case (Figs. 6 and 7) as far as the $p_T$ and $\eta$ distributions are concerned and we choose not to show these distributions for this reason. However, $\cos \theta ^*$ remains an important additional handle to constrain and discriminate different scenarios (albeit at a lower rate due to the leptonic decays of the *Z* boson that we consider here). The distribution of $\cos \theta _h$ essentially discriminates the decays of the transversely polarised *Z* (coefficients *a* and *b*) from the longitudinal one (coefficient *c*). See some examples in Fig. 7.

It is important to add that the background distribution is highly sculpted towards the bulk of signal hypotheses, making further discrimination beyond the aforementioned cases increasingly difficult.

**Fig. 8:**
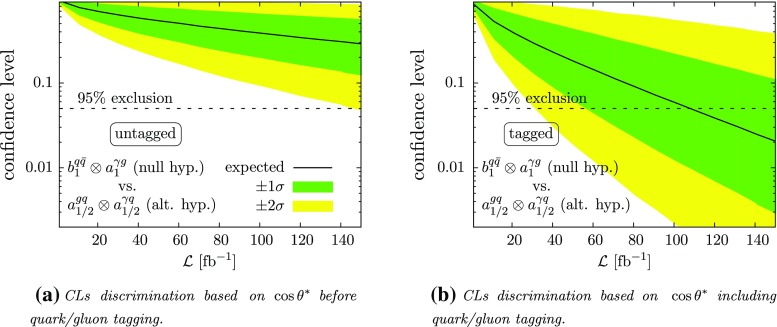
Importance of quark/gluon tagging as a function of integrated luminosity. The overall signal normalisation is aligned with Fig. 9, where we assume a $S/\sqrt{B}=5$ for 300 $\text {fb}^{-1}$ luminosity in the untagged category. This amounts to a signal cross section after cuts of $23.4~\text {fb}$

An additional handle for disentangling the spin hypothesis is through discriminating the production modes. While the transverse momentum distributions of the *Z* boson or the photon give some understanding of the dominant perturbative partonic subprocess, another avenue to further access this information is via identifying, at least approximately (see e.g. the recent Ref. [72]), the quark- or gluon-like character of the leading final state jet. This analysis step, which is entirely complementary to the analysis of angles $\theta _h$ and/or $\theta ^*$, needs to be understood as an additional criterion that is invoked on a final selection that separates signal from background. The drop in signal rate before and after quark/gluon tagging is applied will provide additional power separating integer spin from the ${1\over 2}$ and ${3\over 2}$ hypotheses. This is shown representatively in Fig. 8 for the concrete example of separating the $b^{q\bar{q}}_{1}\otimes a^{\gamma g}_1$ coupling combination as null-hypothesis from the $a^{gq}_{1/2}\otimes a^{\gamma q}_{1/2}$ (alternative) hypothesis. The exclusion limits of Fig. 8 are calculated using the CLs method [73–75] for a binned log-likelihood based on $\cos \theta ^*$ observable in the jet+$\gamma$ channel of Fig. 4, assuming “even” coupling structures.

This particular choice for a representative example is motivated from the overall similarities in the $\cos \theta ^*$ observable, however, with a clear separation of quark- vs. gluon-initiated hard jet. As representative working point for quark-tagging and gluon-rejection, we use efficiencies $(\epsilon ^t_q,\epsilon ^r_g)=(0.5,0.13)$, which have been obtained in Ref. [66] under similar kinematical conditions. Throughout, we include the expected background distributions and the relative reduction of the SM $\mathrm {jet}+\gamma$ production after quark/gluon tagging. For the considered mass range of Eq. (11), the SM continuum production is dominated by processes with a final state quark and the reduction of background cross section is mostly determined by the tagger’s working point. Compared to the jet$+\gamma$ production cross section after selection cuts of $\sim 6.6~\text {pb}$, quark/gluon tagging reduces the cross section by $\sim 47\%$. In Fig. 8b, we add the tagged distributions as a separate category to the likelihood, which can be compared to the “raw” $\cos \theta ^*$ discrimination in Fig. 8a. As can be seen, some of the competing distributions can be excluded to support statistical preference for one particular model for moderate luminosities. This simple hypothesis test which indicates statistical preference between two well-defined hypotheses does not constitute a coupling measurement, but will be the first step in this direction (experimental results related to the Higgs can be found in e.g. [76]).

**Fig. 9:**
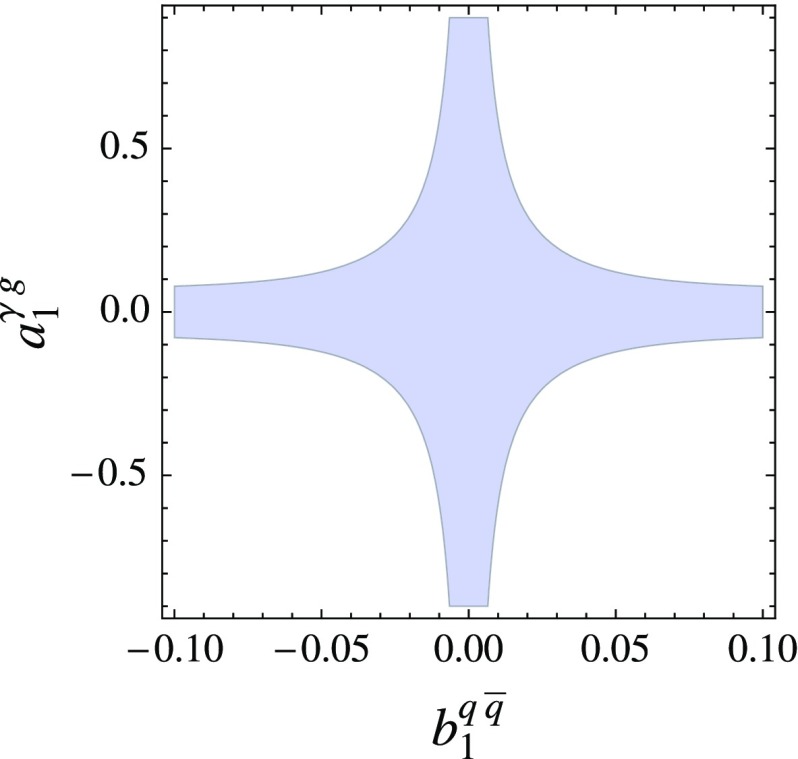
Coupling region from a toy Monte Carlo analysis at $\sqrt{s}=13~\text {TeV}$ for signal cross sections $\sigma <23.4~\text {fb}$, the estimated $5\sigma$ discovery at $\mathcal{{L}}=300~\text {fb}^{-1}$. For details see text

Continuing with this particular example, at higher luminosity, once the $b^{q\bar{q}}_1 \otimes a^{\gamma g}_1$ character is established, the overall rate in excess the background measurement can be used to constrain the couplings of the model. We show this representatively in Fig. 9 for our $M=500~\text {GeV}$ benchmark, showing all parameter combinations that yield a maximum signal cross section of the estimated 5$\sigma$ discovery cross section of $\sim 23.4~\text {fb}$. This MC-based toy extraction also demonstrates that additional information from di-jet measurements is necessary to avoid blind directions, which arise from fitting the narrow width approximation: For large values of e.g. $b^{q\bar{q}}_1$, the jet$+\gamma$ signal cross section scales as a function of $a^{\gamma g}_1$ alone and the limit is saturated by a constraint on this single coupling.

A di-jet constraint, on the other hand, will close the blind direction for large values of $b^{q\bar{q}}_1$. A remaining question is whether the estimated 5$\sigma$ discovery cross section of $\sim 23.4~\text {fb}$ corresponds to a choice of parameters for which our main assumption, i.e. the narrow width approximation is still valid. Scanning the couplings inputting this discovery threshold as a constraint, we can obtain $b^{q\bar{q}}_1$ as a function of $a^{\gamma g}_1$, which then allows one to express $\Gamma _X$ as function of $b^{q\bar{q}}_1$ alone. For our particular benchmark we obtain $\Gamma _X/M\lesssim 0.03$ for $|b^{q\bar{q}}_1|<1$, highlighting the good validity of the narrow width approximation in this case. The branching ratios for the parameter choices that are allowed this way vary between dominant $j\gamma$-like final states for $|b^{q\bar{q}}_1|\ll 1$ to di-jet like final states for $|b^{q\bar{q}}_1|\sim 1$, which again shows the necessity of studying complementary channels in case of a discovery in final states as described in this work.

## Summary

The absence of conclusive hints for new interactions beyond the Standard Model motivates a wider approach to searches for new states that fall within the energy coverage of the LHC or future hadron colliders. Scenarios of QCD-charged new states that could arise in a range of composite Higgs models have been less investigated in the past, and this work presents a detailed investigation of possible $2\rightarrow 2$ scattering processes of such states with jet$+\gamma$ and jet$+Z$ production. This provides the theoretical underpinning for future searches, generalising the current signal modelling of ATLAS and CMS [32–35]. We have based our analysis around a decomposition of the scattering amplitudes into irreducible categories, thereby widening the phenomenological range beyond the constraints of effective field theory. This comes at the price of a strict on-shell formulation of the hard scattering amplitude, which removes a straightforward application of CP-discriminating techniques. However, the phenomenology of the scattering amplitudes proves rich and is observable at the LHC over a broad particle mass range. Naturally, the lack of any observation so far that could be interpreted along the lines of this paper leaves the parameter space vastly bigger than the humble set of angular and kinematic distributions of $2\rightarrow 2$ scattering is capable to constrain. However, we have shown that by adapting angles from the Higgs characterisation program, at least partial insights can be gained into the spin of such a produced state. In particular, we have also demonstrated how advances in jet technology (specifically quark/gluon tagging) can assist the resonance’s discovery or spectroscopy in the future.

## Footnotes

1 Note that the acceptance selections (which are determined by trigger thresholds and mostly finite detector coverage) bias the constructed distribution; see [62] for a detailed discussion in the context of QCD singlets.
